# The link between agriculture and rural food security in the ecoregions of Mexico: path diagrams and underlying dataset

**DOI:** 10.1016/j.dib.2022.108543

**Published:** 2022-08-16

**Authors:** Stéphane Couturier, J. Mauricio Galeana-Pizaña, Daniela Figueroa, Javier Osorno-Covarrubias, Aldo Daniel Jiménez

**Affiliations:** a Laboratorio de Análisis Geo-Espacial (LAGE), Instituto de Geografía, Universidad Nacional Autónoma de México (UNAM), Circuito Exterior, Ciudad Universitaria, Del. Coyoacán, Apdo Postal 20850, Mexico CP 04510, Mexico; b Centro de Ciencias de la Complejidad (C3), UNAM, Mexico City, Mexico; c Centro de Investigación en Ciencias de Información Geoespacial (CENTROGEO), Contoy 137, Col. Lomas de Padierna, Apdo Postal, Mexico, 14240, Mexico; d Instituto de Geografía, Universidad Nacional Autónoma de México (UNAM), Circuito Exterior, Ciudad Universitaria, Del. Coyoacán, Apdo Postal 20850, Mexico CP 04510, Mexico

**Keywords:** Food system, Path diagram, Path relationships, Agricultural system, Smallholder agriculture, Food security, Mexico, Emerging economies

## Abstract

In this research, we build two food systems datasets in Mexico; The first one describes the structure of agricultural production units and the second one describes food security aspects of the rural population in these agricultural production units. We also build a third dataset, consisting of path diagrams and path coefficients (derived from Structural Equation Modeling) that relate the first dataset to the second dataset in the four most populated ecoregions of Mexico. The description of the path models and the insights they bring to the current state of food security in Mexican rural households are detailed in an associated article entitled “Is food security primarily associated with smallholder agriculture or with commercial agriculture?: An approach to the case of Mexico using structural equation modeling”

(https://doi.org/10.1016/j.agsy.2021.103091).

The agricultural variables (in the first dataset) include farm size, destination of the farmer's production, cultivation practice / water management, predominant source of income of the household, land tenure type, crop diversity, agricultural surface expansion, and the presence of forest cover. They are based on the primary data of the full, latest available agricultural census in Mexico and corresponding official land use / land cover data. The second dataset consists of four food security indicators designed and built for the first food security model in Mexico that incorporates food availability, food accessibility and food utilization aspects. They include the Food Self-sufficiency Index (the balance between food production and food consumption), the Food Access Index (inversely related to marginalization), the Entitlement to Public Health Care index, and the Undernutrition Infrequency index (related to hospital sickness records). We provide the path tables and diagrams that describe the links between the agricultural structure and food security. These diagrams provide the first nationwide statistical evidence for the prominent role of smallholder agriculture in rural food security at the national level and at ecoregion scale for a country of the global South.

In order to further investigate the structure of the agricultural production units and their relationships with socio-economic, territorial and landscape data, artificial intelligence (i.e. data mining and machine learning) techniques could be performed on this compendium of datasets. The food security data may stir the development of more food security models in Mexico in relation to other drivers such as consumption habits and non-agricultural activities of rural households.

**Specifications Table**

**Table:** 

Subject	Agricultural Economics.
Specific subject area	Smallholder agricultural structure. Food security.
Type of data	Tables (7) Graphs (diagrams) (4) GIS layer (1)
How data were acquired	The agricultural features are derived from the 2007 agricultural national census (the latest available census at the date of publication of this research), which was acquired at rural neighbourhood level (“Área de control” in Spanish) on a request to the Instituto Nacional de Estadística y Geografía (INEGI [1]). The data are usually available online on a “municipio” level request basis. The land use variables are derived from Land Use and Vegetation Cover vector data acquired online (“El Mapa Digital de México” [2,3]). Non-agricultural complementary variables (education level, road density) were also acquired. The *education level* variable was derived from the demographic and housing 2010 census and acquired online [4]. The *road density* variable was derived from the National Road Network 2017 and acquired online [5]. The food security indicators were derived from official national data as well; The *Food Self-sufficiency Index (FSI)* was derived from the 2007 agricultural census [1] and from the National Chamber for Transformation Industries [6].
	The *Food Access Index (FAI)* was derived from the marginalization index, available on the National Population Council in Mexico website [7]. The percent population entitled to public health care (the Entitlement to Public Health Care index) was also acquired from the national demographic and housing census [4]. Finally, the *Undernutrition Infrequency* index was derived from the 2007-2017 data available in the National Health Ministry website [8].
Data format	1. Raw data: All variables and indices (categorical and continuous values) are released in one Geographic Information System (GIS) vector layer (shapefile) at the national level 2. Secondary data 1: The unstandardized coefficients and standard errors of path analysis are released in the form of tables in this manuscript 3. Secondary data 2: The path diagrams obtained from structural equation modeling are released in the form of figures in this manuscript
Parameters for data collection	The primary data were collected at rural neighbourhood level (« área de control », in Spanish: the smallest spatial unit of the agricultural census [1]) for each neighbourhood that contained rural population. The data were organized for analysis at two scales: the national scale and the ecoregion scale. At ecoregion scale, we only present the data from the four more populated ecoregions of Mexico (Fig. 1) because the number of observations is high and similar in these ecoregions (Table 1). The lower number of observations in the remaining three ecoregions limited the significance of models [9].
Description of data collection	After acquisition of the primary data, ten characteristics of the agricultural systems were computed: 4 categorical indicators and 6 continuous variables. 2 non-agricultural variables were added to conform the dataset of explanatory variables. 4 indices were also computed to conform the food security dataset. Path analysis was applied to link both datasets at the national level and for 4 ecoregions. The resulting diagrams, unstandardized coefficients and standard errors of the path relationships compose the remaining data presented in this compendium.
Data source location	Institutions: INEGI, CONAPO, the National Chamber for Transformation Industries. City/Town/Region: Variables for the entire country, and diagrams for four ecoregions of Mexico [10]: Temperate Sierras, Southern Semi-Arid Highlands, Tropical Wet Ecoregion, Tropical Dry Ecoregion. Country: Mexico
Data accessibility	Hosted in the following website: 10.6084/m9.figshare.19723423.v2 The raw data is also available in GIS format in the following repository: http://seguralimentariamex.igg.unam.mx
Related research article	J.M. Galeana-Pizaña, S. Couturier, D. Figueroa, A. D. Jiménez, Is rural food security primarily associated with smallholder agriculture or with commercial agriculture?: an approach to the case of Mexico using structural equation modeling, Agric. Syst. 290 (2021) 103091. 10.1016/j.agsy.2021.103091

## Value of the Data

• This is the first release of standardized attributes of smallholder agriculture at rural neighbourhood (“área de control”) level in Mexico. The datasets presented provide the most recent and spatially detailed basis for agricultural structure analysis and for food security modeling in Mexico. Understanding the livelihood strategies of smallholders within the major ecogeographical regions of a country is a guideline for the orientation of incentives and necessary subsidies in agricultural policies.
• These data can benefit to: the scientific community specialized in food systems and agricultural economics; decision making bodies in charge of agricultural and environmental policies at local, state and federal levels in Mexico; professionals at the National Health Institute in Mexico. The relationships between food security and rural household livelihood strategies are key to social and public health policies.
• The indicators of smallholder agriculture may be reused to seek relationships with socio-economic, territorial, and environmental data. The food security data are useful as input to future food security models in Mexico in relation to other drivers such as consumption habits, type of food distribution outlets, non-agricultural activities, etc.
• This release allows the immediate use of valuable indicators for further studies on livelihood strategies of smallholder households versus livelihood strategies of large farmers.
• This release allows the immediate use of valuable indices for further studies on drivers of food security among the rural population in Mexico.
• The environmental variables documented in this dataset are unavailable in the official agricultural census; they are key to study environmental impacts of the agricultural system in Mexico and to provide a rationale for agricultural policies towards higher environmental sustainability, including gradually guiding key sectors of the Mexican agriculture to a necessary agroecological transition.

## 1 Data Description

**Fig. 1:**
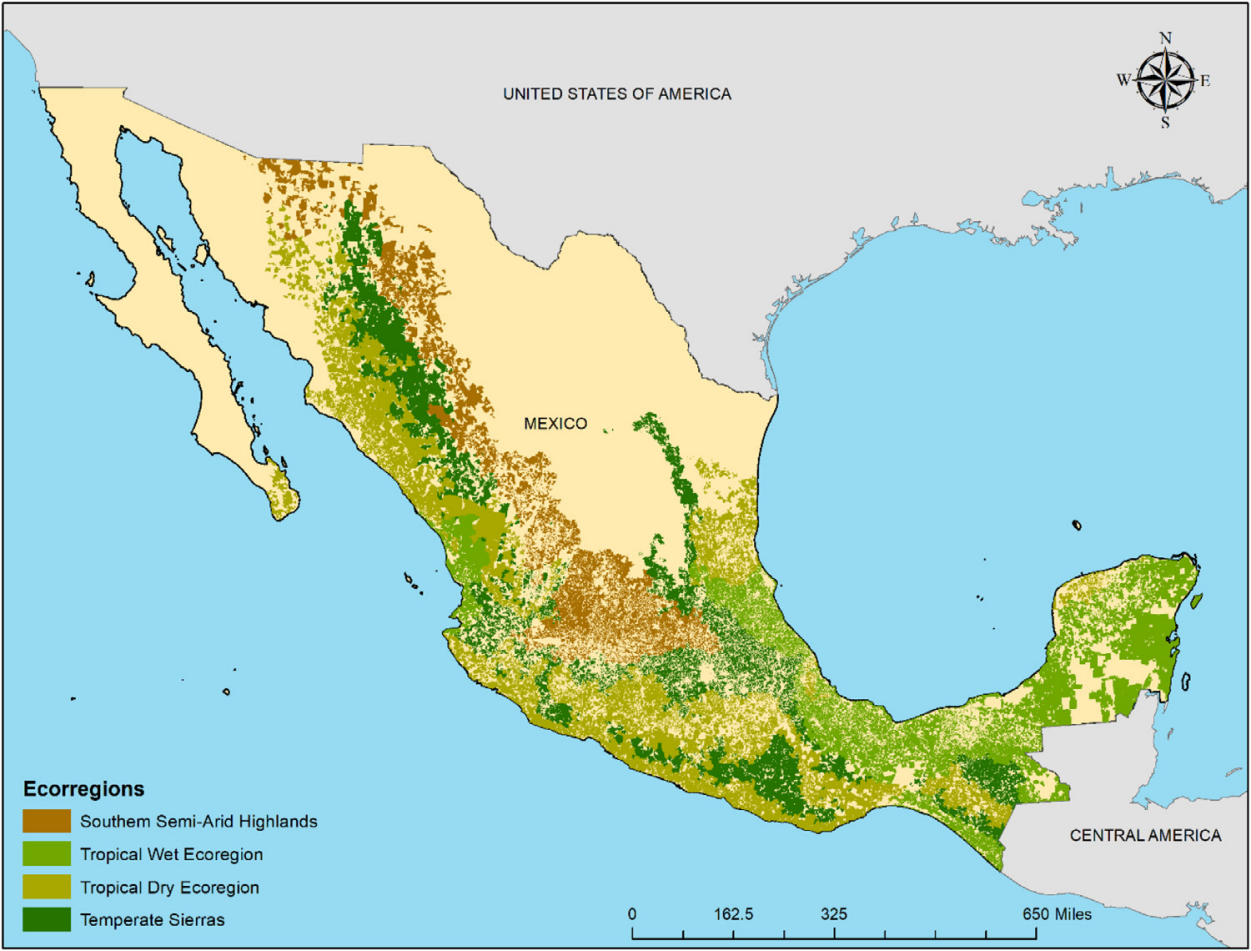
Spatial extension of the dataset where path relationships are presented in this release (all rural neighbourhoods in the four major ecoregions of Mexico [10]). The spatial unit is the rural neighbourhood (“área de control” in Spanish) of the 2007 agricultural census [1]. The number of observations for Southern Semi-Arid Highlands, Tropical Wet Ecoregion, Tropical Dry Ecoregion, and Temperate Sierras are 13,144, 15,836, 14,817, and 15,824, respectively. This figure was adapted from [9] (Fig. 1).

This data release consists of a compendium of three datasets that represents characteristics of the rural population in Mexico (Fig. 1; Table 1). The first dataset comprises nationwide georeferenced features of agricultural production units at the rural neighbourhood scale (Table 2); the second dataset addresses food security of the corresponding rural population (Table 3); the third dataset consists in representations (Table 4, Table 5, Table 6, Table 7 and Fig. 2a–d) of relationships between the two previous datasets according to path analysis (structural equation models, see next section) between the two systems (the agricultural system and the food security system). The information on the two first datasets is disaggregated (see next section for methodological details) at the rural neighbourhood level, the smallest spatial unit of the agricultural census in Mexico (“Área de control” in Spanish, see [1]). The first and second datasets are available at the national level (number of observations: 68,323) and the third dataset is available at ecoregion level for the four most populated ecoregions of Mexico (Fig. 1; Table 1; number of observations: 13,144–15,836, see Fig. 1) through the following URL:

**Table 1:** Bioclimatic features of the four major ecoregions of Mexico (data derived from [11]).

Ecoregion	No. climatic types	Annual rainfall (mm)	Minimun temperature (°C)	Maximun temperature (°C)	Vertebrate species biodiversity
Southern Semi-Arid Highlands	25	200-1500	8 – 10	22 - 24	1000
Temperate Sierras	45	200 - 4500	< - 2	> 28	1980
Tropical Dry Ecoregion	35	50 -2500	12 – 14	> 28	1890
Tropical Wet Ecoregion	19	600 - 4500	14 - 16	26-28	1650

**Table 2:** Description of the agricultural features (explanatory variables), disaggregated at the rural neighbourhood level (“Área de control” in Spanish, see [1]).

Index / Variable	Definition	Data type	Categories	Value Range	Data source
Smallholder index	Farm size range according to the mean per-farmer size of the agricultural parcel (ha) [12].	Categorical	(1) Very small (< 2 ha); (2) Small (2–5 ha); (3) Medium (5–15 ha); (4) Large (15–50 ha); (5) Extensive grazing (>15ha, and pasture area >90%).	1 0.8 0.6 0.4 0.2	INEGI [1]

Subsistence index	Predominant destination type / marketing channel of the farmer's production [13].	Categorical	(1) Subsistence; (2) Mixed (Subsistence & Market commercialization); (3) Market commercialization); (4) Business.	1 0.75 0.5 0.25	INEGI [1]

Rainfed cultivation index	Predominant cultivation practice / water management / pasture management (based on the largest surface per spatial unit) [9].	Categorical	(1) Rainfed system (2) Mixed (Rainfed-irrigated systems) (3) Irrigated system (4) Pasture	1 0.75 0.5 0.25	INEGI [3]

Economic diversification index	Predominant source of income (agricultural activity versus other sources such as international remittances, off farm labour and public assistance programs [14].	Categorical	(1) Pluri-active (non-agricultural activities) (2) Mixed (agricultural and non-agricultural activities) (3) Specialized (agricultural activities)	1 0.66 0.33	INEGI [1]

Land tenure	Fraction of “Ejidal” surface in the rural neighbourhood.	Numerical		0-1	INEGI [1]

	Fraction of “Communal” surface in the rural neighbourhood.	Numerical		0-1	INEGI [1]

Fraction of private surface in the rural neighbourhood.	Numerical		0-1	INEGI [1]

Crop diversity	Shannon index applied to the number of crops and associated cultivated areas [15].	Numerical		0-1	INEGI [1]
Agricultural expansion	Proportion of the land converted to agricultural land in the rural neighbourhood.	Numerical		0-1	INEGI [2,3]

Presence of forest cover	Proportion of forest ecosystems in the rural neighbourhood.	Numerical		0-1	INEGI [3]

**Table 3:** Description of the food security indices (independent variables).

Name of the indicator	Definition	Data type	Data source
Food self-sufficiency index	Normalized difference between the amount of food production and the amount of food consumption [16].	Numerical	INEGI [1,4]. García-Urigüen [6].
Food access index	Inverse of the Marginalization Index [7].	Numerical	INEGI [7].
Entitlement to public health care	Fraction of the population with access to the public health care system.	Numerical	INEGI [4].
Undernutrition infrequency	Inverse of the fraction of the population that was registered with enterogastric related sicknesses and morbidity in public hospitals in 2007 – 2017.	Numerical	Secretaria de Salud de México [8].

**Table 4:** Strength of the path relationship between the ***features of the agricultural production units*** (predictor variables) and the ***food self-sufficiency index***. Unstd Coeff: Unstandardized maximum likelihood path coefficient. Std. Error: Standard Error. Hyphens indicate non selected variables after model re-specification.

	Mexico (national scale)		Temperate Sierras		Southern Semi-Arid Highlands		Tropical Wet Ecoregion		Tropical Dry Ecoregion	
Predictor Variables:	Unstd. Coeffs	Std. Error	Unstd. Coeffs	Std. Error	Unstd. Coeffs	Std. Error	Unstd. Coeffs	Std. Error	Unstd. Coeffs	Std. Coeffs
Smallholder index	0.30^⁎⁎⁎^	0.000	0.18^⁎⁎⁎^	0.001	0.60^⁎⁎⁎^	0.001	0.40^⁎⁎⁎^	0.001	0.10^⁎⁎^	0.000
Subsistence index	-0.10^⁎⁎⁎^	0.000	-0.30^⁎⁎⁎^	0.001	-0.20^⁎⁎⁎^	0.001	-	-	-	-
Rainfed cultivation index	-0.10^⁎⁎⁎^	0.000	0.30^⁎⁎⁎^	0.001	-	-	-	-	-0.1*	0.000
Economic diversification index	-0.10*	0.000	-	-	-0.10*	0.001	-	-	-	-
Land tenure (Ejidal)	0.31^⁎⁎⁎^	0.003	0.32*	0.011	-	-	-	-	-	-
Land tenure (Communal)	-0.07	0.006	-	-	-	-	-	-	-	-
Land tenure (Private)	0.11^⁎⁎⁎^	0.002	-	-	-	-	0.46^⁎⁎⁎^	0.011	-	-
Agricultural expansion	-	-	-	-	0.31^⁎⁎⁎^	0.005	-	-	-	-
Crop diversity	-0.18^⁎⁎⁎^	0.000	0.70^⁎⁎⁎^	0.002	-	-	-	-	-	-
Presence of forest cover	-	-	-	-	-	-	-	-	-	-

Signif. Codes: 0 ‘***’ 0.001 ‘**’ 0.01 ‘*’ 0.05 “.” 0.1 ‘ ‘ 1.

**Table 5:** Strength of the path relationship between the ***features of the agricultural production units*** (predictor variables) and the ***food access index***. Unstd Coeff: Unstandardized maximum likelihood path coefficient. Std. Error: Standard Error. Hyphens indicate non selected variables after model re-specification.

	Mexico (national scale)		Temperate Sierras		Southern Semi-Arid Highlands		Tropical Wet Ecoregion		Tropical Dry Ecoregion	
Predictor Variables:	Unstd. Coeffs	Std. Error	Unstd. Coeffs	Std. Error	Unstd. Coeffs	Std. Error	Unstd. Coeffs	Std. Error	Unstd. Coeffs	Std. Error
Smallholder index	0.87^⁎⁎⁎^	0.004	0.12^⁎⁎⁎^	0.009	0.21^⁎⁎⁎^	0.013	0.47^⁎⁎⁎^	0.006	0.33^⁎⁎⁎^	0.008
Subsistence index	-0.72^⁎⁎⁎^	0.003	0.80^⁎⁎⁎^	0.006	-0.37^⁎⁎⁎^	0.008	-0.35^⁎⁎⁎^	0.005	-0.99^⁎⁎⁎^	0.006
Rainfed cultivation index	-0.26^⁎⁎⁎^	0.002	-	-	-0.24^⁎⁎⁎^	0.006	-0.07*	0.003	-0.38^⁎⁎⁎^	0.004
Economic diversification index	0.51^⁎⁎⁎^	0.004	0.63^⁎⁎⁎^	0.006	-0.36^⁎⁎⁎^	0.010	0.14*	0.006	0.60^⁎⁎⁎^	0.009
Land tenure (Ejidal)	0.33^⁎⁎⁎^	0.058	-	-	0.61^⁎⁎⁎^	0.130	-	-	0.61^⁎⁎^	0.202
Land tenure (Communal)	-	-	-	-	0.72*	0.398	-0.82*	0.402	-0.37*	0.591
Land tenure (Private)	0.53^⁎⁎⁎^	0.038	-	-	-	-	0.21*	0.094	0.31^⁎⁎⁎^	0.072
Agricultural expansion	-0.29^⁎⁎⁎^	0.039	-0.30^⁎⁎⁎^	0.061	0.15*	0.068	-0.20^⁎⁎⁎^	0.037	-0.97*	0.053
Education level	0.22^⁎⁎⁎^	0.005	0.44^⁎⁎⁎^	0.010	0.20^⁎⁎⁎^	0.012	0.25^⁎⁎⁎^	0.008	0.21^⁎⁎⁎^	0.010
Road density	0.50^⁎⁎⁎^	0.004	0.42^⁎⁎⁎^	0.008	-0.94^⁎⁎⁎^	0.009	0.18*	0.008	-0.23^⁎⁎^	0.008

Signif. Codes: 0 ‘***’ 0.001 ‘**’ 0.01 ‘*’ 0.05 “.” 0.1 ‘ ‘ 1.

**Table 6:** Strength of the path relationships between the ***features of the agricultural production units*** (predictor variables) and the ***Entitlement to Public Health Care*** (a food utilization indicator). Unstd Coeff: Unstandardized maximum likelihood path coefficient. Std. Error: Standard Error. Hyphens indicate non selected variables after model re-specification.

	Mexico (national scale)		Temperate Sierras		Southern Semi-Arid Highlands		Tropical Wet Ecoregion		Tropical Dry Ecoregion	
Predictor variables:	Unstd. Coeffs	Std. Error	Unstd. Coeffs	Std. Error	Unstd. Coeffs	Std. Error	Unstd. Coeffs	Std. Error	Unstd. Coeffs	Std. Error
Smallholder index	0.12^⁎⁎⁎^	0.004	0.17^⁎⁎⁎^	0.011	-	-	0.86^⁎⁎⁎^	0.009	0.11^⁎⁎⁎^	0.009
Subsistence index	-0.25^⁎⁎⁎^	0.003	-	-	-	-	-	-	-0.94^⁎⁎⁎^	0.007
Rainfed cultivation index	-	-	-	-	-	-	0.12*	0.005	0.1*	0.005
Economic diversification index	0.7^⁎⁎⁎^	0.004	0.88^⁎⁎⁎^	0.088	0.77^⁎⁎⁎^	0.008	0.41^⁎⁎⁎^	0.010	0.58^⁎⁎⁎^	0.010
Land tenure (Ejidal)	-	-	-	-	-	-	-0.44^⁎⁎⁎^	0.276	0.72^⁎⁎^	0.225
Land tenure (Communal)	-	-	-	-	-	-	-	-	-	-
Land tenure (Private)	0.16^⁎⁎⁎^	0.039	0.24*	0.089	-0.16*	0.069	0.34*	0.151	0.16*	0.080
Education level	-0.71^⁎⁎⁎^	0.005	-0.62^⁎⁎⁎^	0.013	-0.68^⁎⁎⁎^	0.010	-0.75^⁎⁎⁎^	0.013	-0.74^⁎⁎⁎^	0.011
Road density	0.85^⁎⁎⁎^	0.005	0.46^⁎⁎⁎^	0.011	-	-	0.27^⁎⁎⁎^	0.013	-	-

Signif. Codes: 0 '***' 0.001 '**' 0.01 '*' 0.05 "." 0.1 ' ' 1.

**Table 7:** Strength of the path relationship between the ***features of the agricultural production units*** (predictor variables) and ***Undernutrition Infrequency*** (a food utilization indicator). Unstd Coeff: Unstandardized maximum likelihood path coefficient. Std. Error: Standard Error. Hyphens indicate non selected variables after model re-specification.

	Mexico (national scale)		Temperate Sierras		Southern Semi-Arid Highlands		Tropical Wet Ecoregion		Tropical Dry Ecoregion	
Predictor Variables:	Unstd. Coeffs	Std. Error	Unstd. Coeffs	Std. Error	Unstd. Coeffs	Std. Error	Unstd. Coeffs	Std. Error	Unstd. Coeffs	Std. Error
Smallholder index	0.32^⁎⁎⁎^	0.000	0.46^⁎⁎⁎^	0.001	0.50*	0.003	0.40^⁎⁎⁎^	0.001	0.40^⁎⁎⁎^	0.001
Subsistence index	-	-	-0.17^⁎⁎^	0.000	-	-	0.10^⁎⁎^	0.000	-	-
Rainfed cultivation index	-	-	-	-	0.30^⁎⁎^	0.001	0.10^⁎⁎^	0.000	-	-
Economic diversification index	-	-	-	-	-	-	-	-	-0.30^⁎⁎^	0.001
Agricultural expansion	-0.54^⁎⁎⁎^	0.002	-0.33^⁎⁎⁎^	0.004	-0.36^⁎⁎^	0.013	-0.57^⁎⁎⁎^	0.004	-0.58^⁎⁎⁎^	0.006
Crop diversity	0.33^⁎⁎⁎^	0.000	0.50^⁎⁎⁎^	0.001	0.22^⁎⁎⁎^	0.002	-	-	0.70^⁎⁎⁎^	0.001
Education level	0.38^⁎⁎⁎^	0.000	0.20*	0.001	0.17^⁎⁎⁎^	0.002	0.40^⁎⁎⁎^	0.001	0.50^⁎⁎⁎^	0.001
Road density	-	-	0.17*	0.001	-	-	-0.30^⁎⁎⁎^	0.001	-	-

Signif. Codes: 0 '***' 0.001 '**' 0.01 '*' 0.05 "." 0.1 ' ' 1

**Fig. 2a:**
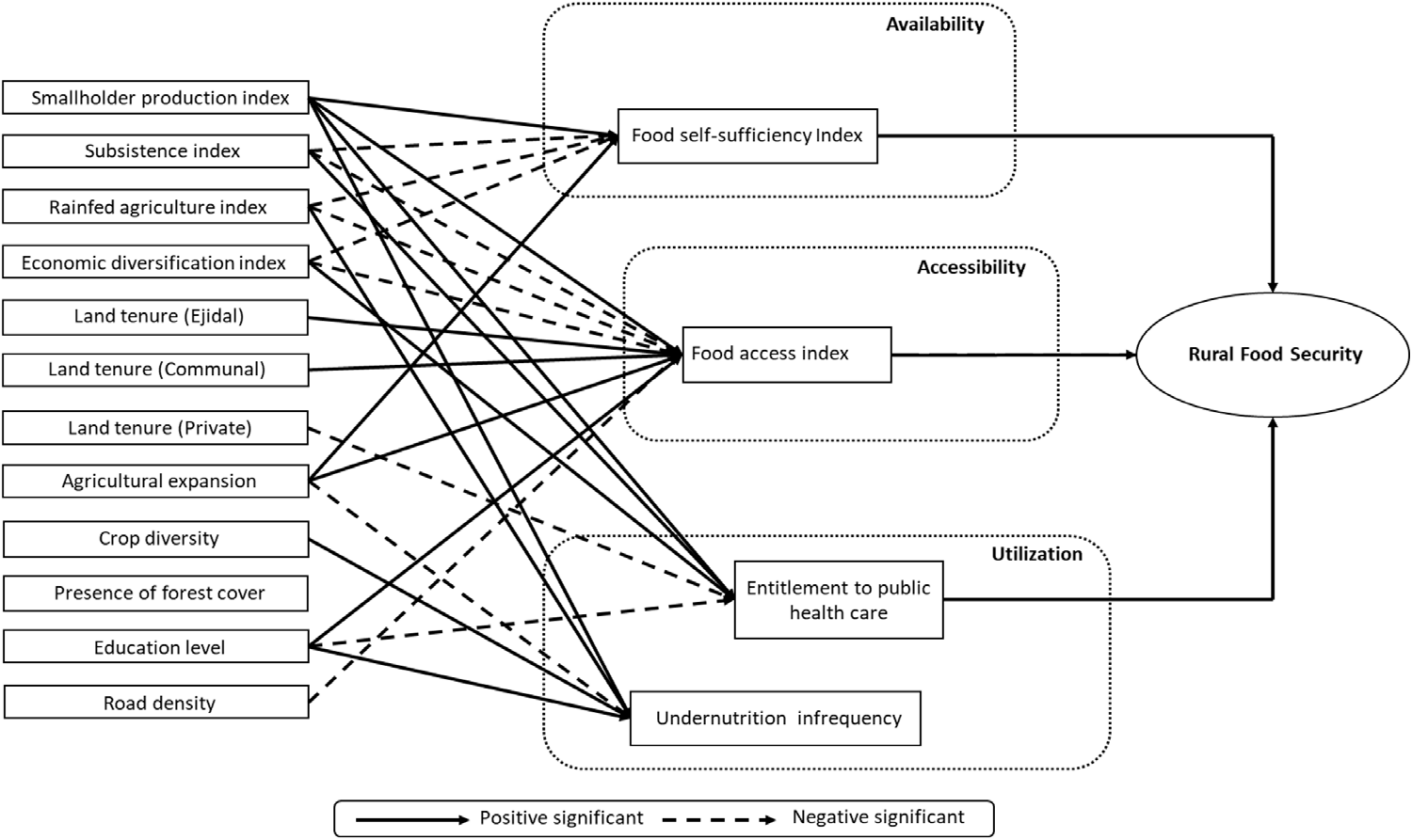
Diagram showing path relationships between aspects of food security and features of the agricultural production units (Southern Semi-Arid Highlands).

**Fig. 2b:**
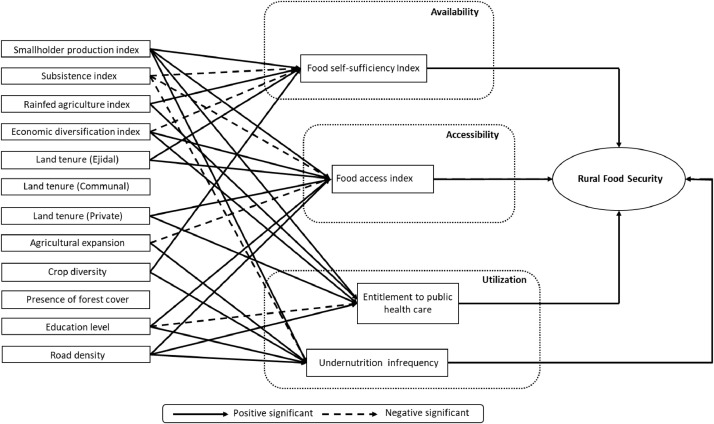
Diagram showing path relationships between aspects of food security and features of the agricultural production units (Temperate Sierras).

**Fig. 2c:**
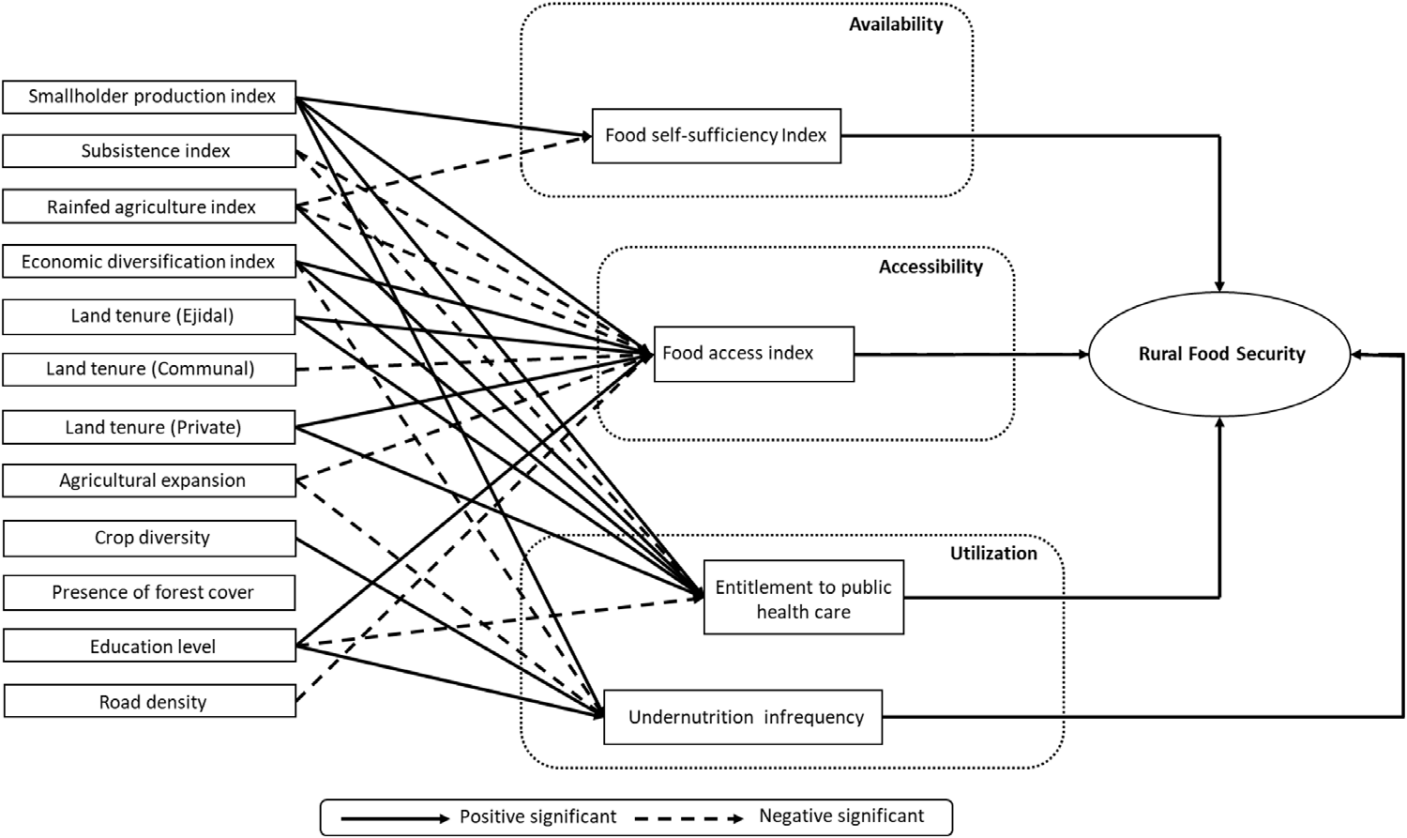
Diagram showing path relationships between food security and features of the agricultural production units (Tropical Dry ecoregion).

**Fig. 2d:**
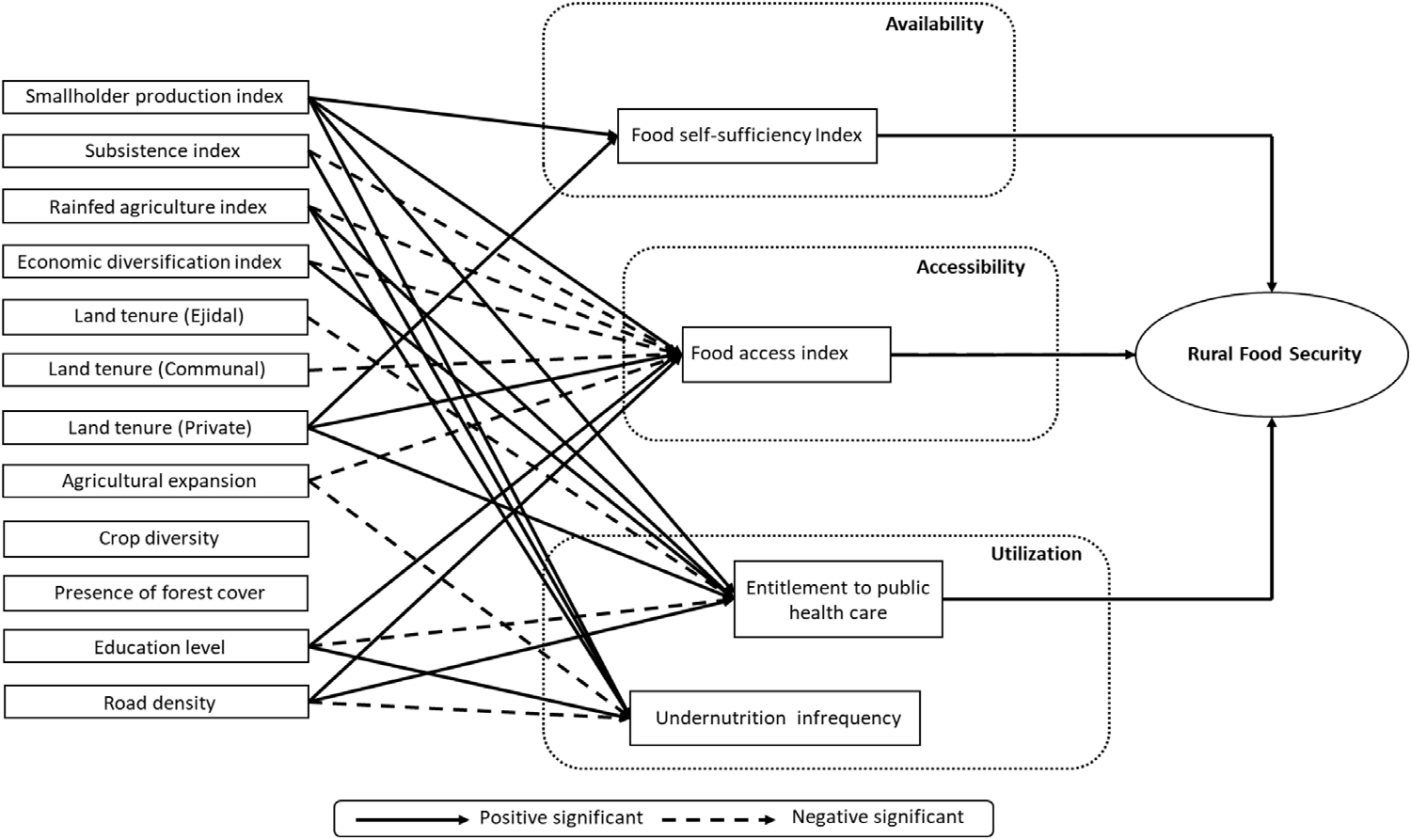
Diagram showing path relationships between food security and features of the agricultural production units (Tropical Wet ecoregion).

https://doi.org/10.6084/m9.figshare.19723423.v2

A selection of the mentioned variables and indices were mapped and made available online in the following Geonode spatial data infrastructure:

http://seguralimentariamex.igg.unam.mx/maps/?limit=5&offset=0

In short, the set of data presented in this compendium is the following:

Fig. 1: Spatial extension of the agriculture and food security datasets where path relationships are presented in this data release
Fig. 2: Diagrams of path relationships between the agricultural systems (first dataset), food security indices (second dataset) and the overall concept of "rural food security ". (a) Southern Semi-Arid Highlands; (b) Temperate Sierras; (c) Tropical Dry ecoregion;
Table 1: The four major ecoregions of Mexico (bioclimatic features)
Table 2: Features of the agricultural production units (first dataset).
Table 3: Food security indices (second dataset).
Table 4: Unstandardized path coefficients linking Food self Sufficiency to predictor variables of the agricultural systems.
Table 5: Unstandardized path coefficients linking Food Access to predictor variables of the agricultural systems.
Table 6: Unstandardized path coefficients linking Entitlement to Public Health Care to predictor variables of the agricultural systems.
Table 7: Unstandardized path coefficients linking Undernutrition Infrequency to predictor variables of the agricultural systems.

### 1.1 Raw Data

The raw data in this data release is a nationwide Geographic Information System (GIS) layer (shape format) comprising georeferenced features of the agricultural production units (indicators and variables) and food security indices of the corresponding rural population. This nationwide dataset is disaggregated at the rural neighbourhood level ("área de control", the smallest spatial unit of the agricultural census in Mexico) and yields 68,323 data units (number of observations = 68,323).

The fields include 15 features of the agricultural production units, 4 food security indices and the ecoregion label (a total of 20 fields of information).

This raw data is available at:

https://doi.org/10.6084/m9.figshare.19723423.v2.

## 2 Experimental Design, Materials and Methods

In this research, major features of the agricultural production units are considered for the characterization of the agricultural systems in Mexico (Table 2). Based on a selection of variables from the latest agricultural census and other official national sources, indicators were built to differentiate smallholder agriculture (high values) from commercial agriculture (low values) in the vector data. A major rationale for the design of this dataset was to offer the opportunity to investigate possible causal relationships between the agricultural structure (see next section “The agricultural features as explanatory variables”) and important societal challenges such as the reduction of poverty, the environmental sustainability of food production, and food security among the population.

In our case, we present potential relationships that could explain patterns of rural food security in Mexico; Food security indices are acquired (see section “The food security indices as independent variables” and Table 3) and path analysis is used to extract relationships between the agricultural structure and food security in Mexico (see section “the structural equation model and path relationships” and Fig. 2 a–d).

### 2.1 The agricultural Features as Explanatory Variables

The first dataset includes a total of 12 features of the rural neighbourhood: five agricultural indicators (smallholder index, subsistence index, rainfed cultivation index, economic diversification index, and crop diversity), three land tenure variables (ejidal, communal and private land surfaces), two land use variables (agricultural expansion, presence of forest cover), and two non-agricultural complementary variables (education level, and road density). The agricultural features are defined in Table 2.

The five agricultural indicators are designed to vary along a gradient that distinguishes smallholder agriculture (high values) from commercial agriculture (low values) and largely relate to the latest agricultural census in Mexico (2007), the most detailed and most reliable source of information on the structure of the agricultural systems [1]. The smallholder index refers to the mean farm size, the subsistence index to the destination of the produces; the rainfed cultivation index relates to water management, the economic diversification to the non-agricultural source of income (Table 2). The reader may explore the spatial distribution of these five agricultural indices on our online repository at http://seguralimentariamex.igg.unam.mx/maps/.

The land use variables are derived from the nationwide official land use land cover cartography in Mexico [2,3]; the agricultural expansion corresponds to the percent increase in agricultural land between 2007 and 2014 and the presence of forest cover is the fraction of forested ecosystems in 2007. The complementary variables also provide relevant information on the agricultural systems in rural neighbourhoods; The education level corresponds to the mean value of the education category of farmers according to the population and housing census of 2010 [4]. A data aggregation via Geographic Information System (GIS) was necessary to translate the Education level per locality (point vector) to the extent of the encompassing rural neighbourhood (polygon vector). The road density variable is the kernel density value of the national road network in each rural neighbourhood in 2017 [5].

### 2.2 The Food Security Indices as Independent Variables

The second dataset consists of four food security indices (Table 3) designed and built for the first food security model in Mexico that incorporates food availability (represented by the Food Self-sufficiency index), food accessibility (represented by the Food access index), and food utilization (represented by the Entitlement to Public Health Care and the Undernutrition Infrequency indices) aspects [9].

A high Food Self-sufficiency Index expresses the excess of production of major crops and livestock (maize, wheat, rice and bean crops; bovine and porcine livestock) with respect to their average consumption in the rural neighbourhood and equates the normalized difference between food production (derived for year 2007 from [1]) and food consumption (derived for year 2010 from [4,6]). This index was proposed by Galeana-Pizaña et al. [16]. The Food Access Index relates to the monetary capacity of households to purchase food and is inversely related to the marginalization index [7]. Entitlement to Public Health Care is an indicator of the access to the nutritional education, prevention from micronutrient deficiency and other health services provided by the public health care infrastructure in rural areas. The index equates the fraction of the population registered under either of the national public health care schemes in Mexico (“IMSS”, “ISSSTE” or “Seguro Popular”) in 2010 [1]. The frequency of enterogastric sicknesses and of morbidity is an indicator of proper food utilization. We extracted the fraction of the population registered in hospitals with enterogastric related sicknesses and morbidity cases in all rural neighbourhoods during 2007–2017 [8]. We considered the hospitals in all localities within the rural neighbourhood for the account of sickness and morbidity cases. The Undernutrition Infrequency was approached using the inverse of this fraction.

The reader may explore the spatial distribution of three food security indices (ex.: Food Self-sufficiency Index, Food Access Index and Undernutrition Infrequency) on our online repository at http://seguralimentariamex.igg.unam.mx/maps/.

### 2.3 The Structural Equation Models and Path Relationships

Structural Equation Modeling (SEM), also known as covariance structure analysis, tests the degree of adjustment between multivariate datasets (assumed with normal distribution) according to a predefined hypothetical model [17]. Based on the previous knowledge on relationships between variables, the hypothetical model is represented by a network-shaped mental map [18]. The extraction of relative strengths of relationships via SEM is based on factor analysis and linear regression [19]. In a first step, a set of a priori specifications (potentially meaningful relationships) is attempted and the SEM model is run. If the fit of the model to the data is sub-optimal, the set of specifications is modified, ruling out non-significative relationships among variables [19], and the SEM model is run again. Eventually, a set of specifications of the model obtains a good fit to the data. The results of the SEM model are represented as path diagrams and path coefficients that express the relative strengths of relationships, potentially useful for the understanding of causal pathways between systems [18].

In our case, predefined potential relationships were already defined between the agricultural system and a latent (non-observed) food security variable using an intermediate set of observed food security indices in Mexico [9] (see Fig. 2). The results of the SEM model and the corresponding path diagram at the national scale were published by Galeana Pizaña et al. [16], including interpretations of the results in terms of potential causal pathways. In this dataset release, we present the results (path diagrams and unstandardized path coefficients) of four re-specified SEM models for each of the four most populated ecoregions of Mexico (Fig. 1) with a good fit according to root mean square error of approximation (RMSEA) and comparative fit index (CFI). The four SEM models were built using the confirmatory factor analysis (CFA) within the LAVAAN R package [20]. The vector data were not standardized among ecoregions because the numbers of observations are similar (Fig. 1).

Highly significant (*p* < 0.001) unstandardized path coefficientsmark the strong relationships that exist between agricultural features and a particular food security aspect (Table 4, Table 5, Table 6, Table 7). In each table, the unstandardized path coefficients are reported at national and ecoregion levels (Table 4, Table 5, Table 6, Table 7). The path diagrams (Fig. 2 a–d) are synthetic, graphical representations of these relationships, potentially useful for regional stakeholders in agricultural, food and health public policy in the respective ecoregions of Mexico.

Additionally, the exploration of the spatial pattern of these relationships may be explored in this dataset release by the public and by the scientific community, through an online repository of this data release at http://seguralimentariamex.igg.unam.mx/maps/.

## Ethics Statement

The dataset and manuscript presented here were derived from genuine, original scientific work and was not submitted for publication nor published elsewhere. A dataset complementary to (but different from) the dataset of this manuscript (obtained with the same method) has been published in [9].

## CRediT authorship contribution statement

**Stéphane Couturier:** Investigation, Conceptualization, Writing – original draft, Supervision. **J. Mauricio Galeana-Pizaña:** Conceptualization, Methodology, Funding acquisition. **Daniela Figueroa:** Software, Project administration. **Javier Osorno-Covarrubias:** Visualization, Data curation. **Aldo Daniel Jiménez:** Investigation, Conceptualization.

## Declaration of Competing Interest

The authors of this manuscript declare that they have no known competing financial interests or personal relationships which have or could be perceived to have influenced the work reported in this article. The research underlying this study received funds from CONACYT (the Public Research Fund Agency in Mexico) and from UNAM (Universidad Nacional Autónoma de México). In particular, the CONACYT funded project 2015-01-687: "Development, optimization and implementation of novel technologies in the molecular and cartographic domains for transgene and herbicide monitoring in Mexico towards an integral strategy and perspective for biosecurity"; the CONACYT funded project A1-S-34633 “De la caracterización química y molecular al aprovechamiento sustentable de especies silvestres de Lupinus”, the CONACYT funded project LN-CONACYT-2021-315858 “Laboratorio Nacional de Observación de la Tierra (LANOT) 2021”, the Geography Institute UNAM supported project “Estimación robusta de tasas de deforestación en apoyo al Sistema Satelital de Monitoreo Forestal (SaMoF)”, the UNAM funded project PAPIIT IN302417 "Food security versus environmental protection: design of a national cartographic platform for a multiscalar analysis of their compatibility"; and UNAM funded project PAPIIT IN302720 "Resilience and livelihood strategy of rural households of the coast of Oaxaca".

## Data Availability

The link between agriculture and rural food security in the ecoregions of Mexico: path diagrams and underlying datasets. (Original data) (Figshare).
